# Antithrombotic effect and action mechanism of *Salvia miltiorrhiza* and *Panax notoginseng* herbal pair on the zebrafish

**DOI:** 10.1186/s13020-020-00316-y

**Published:** 2020-04-16

**Authors:** Shi-Jun Yin, Ying-Qing Luo, Cong-Peng Zhao, Hua Chen, Zhang-Feng Zhong, Shengpeng Wang, Yi-Tao Wang, Feng-Qing Yang

**Affiliations:** 1 School of Chemistry and Chemical Engineering, Chongqing University, Chongqing, 401331 China; 2 School of Life Sciences, Chongqing University, Chongqing, 401331 China; 3 State Key Laboratory of Quality Research in Chinese Medicine, Institute of Chinese Medical Sciences, University of Macau, Macao, China

**Keywords:** *Salvia miltiorrhiza*, *Panax notoginseng*, Zebrafish, Phenylhydrazine, Thrombosis model

## Abstract

**Background:**

*Salvia miltiorrhiza* (Danshen, DS) and *Panax notoginseng* (Sanqi, SQ) are famous traditional Chinese herbs, and their herbal pair (DS–SQ) has been popular used as anti-thrombotic medicines. However, there is still a lack of sufficient scientific evidence to illustrate the optimum combination ratio of these two herbs as well as its action mechanisms. The purpose of this study is to investigate the anti-thrombotic effects of DS–SQ on zebrafish and explore its possible action mechanism.

**Methods:**

Firstly, the chemical components in DS–SQ extract were analyzed by LC–ESI–MS/MS. Then, a phenylhydrazine (PHZ)-induced zebrafish thrombosis model was developed for evaluating the anti-thrombotic effects of DS–SQ extracts with different combination ratios and their nine pure compounds. Followed, Real-time quantitative PCR (RT-qPCR) assays were performed to investigate the potential antithrombotic mechanisms of DS–SQ.

**Results:**

Thirty-three components were tentatively identified by LC–MS analysis. DS–SQ at the ratio of 10:1 presented the best anti-thrombotic effect, and rosmarinic acid, lithospermic acid and salvianolic acid B of DS showed good anti-thrombotic activity on zebrafish thrombosis model. The RT-qPCR assays indicated that DS–SQ (10:1) could cure the PHZ-induced thrombosis by downregulating the expression of *PKCα*, *PKCβ*, *fga*, *fgb*, *fgg* and *vWF* in zebrafish.

**Conclusions:**

DS–SQ with the combination ratio of 10:1 showed optimum anti-thrombotic effect on PHZ-induced zebrafish thrombosis model, which provided a reference for reasonable clinical applications of DS–SQ herbal pair.

## Background

According to the WHO World Health Statistics 2018, an estimated 17.9 million deaths accounting for 44% of noncommunicable diseases (NCDs) occurred due to the cardiovascular diseases [1]. Thrombosis, which plays an important role in cardiovascular disease, seriously threatens human health and life [2, 3]. Currently, the most commonly used anti-thrombotic drugs are aspirin, warfarin and heparin, but they are accompanied by bleeding and drug resistance [4, 5]. Complex pathological conditions such as thrombosis require combinational therapies that can act on multiple biological targets to efficiently manage the underlying mechanistic pathways [6]. Considering that traditional Chinese medicines (TCMs) have the advantages of limited adverse effects, significant efficiency and having active-target diversity, therefore, TCMs become an important research direction to search for anti-thrombotic drugs [7].

Zebrafish, as an animal model has many advantages including rapid propagating and high homology with human genes, has been successfully applied in developmental biology, genetics and human diseases studies [8–10]. In fact, zebrafish are especially suitable for the study of thrombus due to ease observation of circulation system without invasive methods and its good response to anti-coagulant drugs commonly used in clinical treatment [11, 12]. From previous reports, the arachidonic acid (AA)/FeCl_3_-induced zebrafish thrombosis model had been used for evaluation of antithrombotic activity of rosmarinic acid and p-coumaric acid in Danhong injection [13] and natural terpenoid glycosides from the leaves of *Crataegus pinnatifida* [14].

*Salvia miltiorrhiza* (Danshen, DS) and *Panax notoginseng* (Sanqi, SQ), as the traditional medicinal materials that can be used in healthy food, have been focused an increasing number of attentions [15, 16]. The Danshen–Sanqi herbal pair (DS–SQ) has been popular used as the Chinese herbal medicine for prevention and treatment of cardiovascular diseases, including stroke, myocardial infarction and angina pectoris [17]. Recently, DS–SQ was reported having a variety of biological and physiological effects such as protective effects on cell survival in the human cardiovascular endothelial [18], anti-inflammatory activity [19] and inhibition of retina cell apoptosis [20]. A study by Yue et al. revealed that DS–SQ showed cardio-protective effects against ischemia–reperfusion injury [21]. Liu et al. confirmed that DS–SQ with a combination ratio of 10:3 could markedly inhibit platelet aggregation and adhesion in normal rabbit [22]. Although DS–SQ showed the anti-thrombotic activity, there is still a lack of sufficient scientific evidence to illustrate the optimum combination ratio of these two herbs as well as its action mechanisms. Thus, in the present study, taking advantage of transparent zebrafish larvae, a phenylhydrazine (PHZ)-induced zebrafish thrombosis model was developed to evaluate the anti-thrombotic activity of DS–SQ and their nine compounds, and the underlying action mechanism was also explored.

## Materials and methods

### Chemicals and reagents

DS (Lot: 171001) and SQ (Lot: 180304) materials were purchased from Xinyitang Co., Ltd. (Chongqing, China) and were identified as the dried roots and rhizomes of *Salvia miltiorrhiza* and *Panax notoginseng*, respectively, by Professor Feng-Qing Yang of Chongqing University. All the samples were deposited at the Pharmaceutical Engineering Laboratory in School of Chemistry and Chemical Engineering, Chongqing University, Chongqing, China. Danshensu (DSS), protocatechuic acid (PRAC), rosmarinic acid (MA), lithospermic acid (LA), salvianolic acid B (SAB) and salvianolic acid A (SAA) were purchased from PureChem-Standard Co., Ltd. (Chengdu, China). Protocatechualdehyde (PRAL), ginsenoside Rg1 (GRg1) and ginsenoside Rb1 (GRb1) were purchased from Yuanye Biotechnology Co., Ltd. (Shanghai, China). Phenylhydrazine (PHZ) was purchased from Shanghai Titan Scientific Co., Ltd. (Shanghai, China). Aspirin (ASP) was from Chengdu KeLong Co., Ltd. (Chengdu, China). O-dianisidine was from Chongqing Golden magpie Science & Technology Co., Ltd. (Chongqing, China). RNA Extraction Kit and SYBR qPCR Master Mix were purchased from Nanjing Vazyme BioTech Co., Ltd. (Nanjing, China). IScript™ cDNA Synthesis Kit was purchased from Chengdu BIO-BRI Science & Technology Co., Ltd. (Chengdu, China). Acetonitrile (ACN) and formic acid (FA) were of HPLC-grade and obtained from Beijing InnoChem Science & Technology Co., Ltd. (Beijing, China). All of the experimental water was purified by a water purification system (ATSelem 1820A, Antesheng Environmental Protection Equipment Co., Ltd., Chongqing, China).

### Instruments and conditions

An electrospray ionization-mass spectrometer (ESI–MS) consisting of a Shimadzu 8060 Triple-Quadruple mass spectrometer (Shimadzu, Kyoto, Japan), coupled with a HPLC system was used for the chemical analysis of DS–SQ extract. The mobile phase was consisted of solvent A (0.1% FA aqueous solution) and solvent B (ACN) at a flow rate of 0.5 mL/min. A gradient elution was: 5–10% B at 0–2 min, 10–34% B at 2–20 min, 34–41% B at 20–35 min, 41–50% B at 35–60 min, 50–57.5% B at 60–70 min, 57.5–65% B at 70–90 min, 65–95% B at 90–110 min, 95–5% B at 110–115 min, 5% B at 115–120 min. A 10 μL of sample was injected into an Agilent Zorbax SB-Aq column (250 × 4.6 mm, 5 μm), which was maintained at 30 °C, for separation.

The ESI–MS conditions were as follows: drying gas pressure, 100 MPa; curved desolvation line (CDL) voltage, constant level; interface voltage, 1.4 kV; nebulizing gas flow rate, 3 L/min; detector voltage, 1.40 kV; CDL temperature, 235 °C; block heater temperature, 400 °C; and vacuum, 1.9 × 10^−2^ Pa. The mass spectra were recorded in simultaneous positive and negative ionization full-san mode (*m/z* 100–2000). The ion accumulation time was set at 100 ms and the collision energy of collision induced dissociation (CID) was set at 50%. Data acquisition and processing were performed with the LC–MS solution version 1.1 software (Shimadzu).

### Sample preparations

The dried roots of DS and SQ were comminuted and passed through a 50-mesh sieve. Powders of 6.4 g DS and 6.4 g SQ were refluxed with 85 mL of 70% ethanol in water at 70 °C for 3 h, respectively. Then, the extracts were rotationally evaporated at 50 °C and the concentrated solution were dried in the vacuum at 50 °C for 48 h. The extraction yields of DS and SQ were 28.6% and 7.8%, respectively. The DS and SQ extracts were dissolved with 5% ACN at the proportion of DS to SQ (1:1) (final concentration of about 1 mg/mL). After filtered through a 0.45 μm membrane filter, the solution was injected into the HPLC system for LC–MS analysis. The stock solution of DS, SQ, DSS, PRAC, MA, LA, SAB, SAA, PRAL, GRg1, GRb1 and ASP were prepared in ultra-pure water at a concentration of about 1 mg/mL, and then diluted with water as required for zebrafish assays.

### Zebrafish maintenance and embryo collection

AB strain zebrafish were obtained from Shanghai Gene-Bio Co., Ltd. Zebrafish were raised and kept according to the Institutional Animal Care and Westerfield’s method [23]. Zebrafish were maintained at 28 °C in a flow-through system with 14 h light/10 h dark cycle and were fed three times per day. Embryos were obtained via natural spawning. Two pairs of male and female zebrafish were selected for incubation and the embryos were collected and maintained in an incubator at 28 °C for 4 days. The experimental schemes involving conscious animals were approved by the Institutional Animal Ethical Committee of Chongqing University and were conducted in accordance with the Guide for the Care and Use of Laboratory Animals of the National Institute of Health (Publication no. 80-23, revised 1996).

### The exposure experiment of zebrafish larvae

The 4-days post fertilisation (dpf) zebrafish larvae were randomly placed in a 12-well plate containing 5 larvae per well. The larvae were exposed with 1.5 μM PHZ and sample solutions [G1, 25 μg/mL of aspirin; groups G2–G12, 100 μg/mL of DS–SQ with different ratios (10:0, 10:1, 10:2, 10:3, 10:4, 1:1, 4:10, 3:10, 2:10, 1:10, 0:10); groups G13–G16, 10:1 of DS–SQ with different concentrations (12.5, 25, 50 and 100 μg/mL); groups G17–G25, 25 μg/mL of different components (DSS, PRAC, PRAL, MA, LA, SAB, SAA, GRg1 and GRb1)], respectively. The control group G26 was treated with ultra-pure water and the model group G27 was adopted for 1.5 μM PHZ. After incubating in an incubator at 28 °C for 12 h, all the incubation solutions were discarded and the zebrafish were stained with o-dianisidine dye liquor [24] for 15 min in the dark at 28 °C. Then, the zebrafish were rapidly washed by dimethylsulfoxide (DMSO) three times. To assess the anti-thrombosis effect of corresponding groups, the thrombus of the caudal vein and heart of zebrafish larvae were observed and photographed under BM2000 biological microscope (Nanjing Jiangnan Novel Optics Co., Ltd). The dyeing area of heart (S) was quantified by Image-pro Plus 6.0. The anti-thrombotic effect of different groups were evaluated based on the following formula [25]: Thrombosis inhibition percentage (%) = [S(drug) – S(model)]/[S(control) – S(model)] × 100%.

### Gene expression analysis

Gene expression analysis was carried out according to a modified procedure that was reported before [26, 27]. In brief, the zebrafish larvae were divided into three groups (G28–G30) (30 larvae per group). After exposure in the 1.5 μM PHZ and 100 μg/mL DS–SQ (10:1), zebrafish larvae were washed with water for three times, and were homogenized after hatching for 12 h. Total RNAs were isolated from dissociated larvae using RNA Extraction Kit (Vazyme). Then, RNAs were converted to single-strand cDNA with IScript™ cDNA Synthesis Kit (BIO-BRI). Real-time quantitative PCR (RT-qPCR) was carried out for gene expression using the SYBR qPCR Master Mix (Vazyme), and was performed in triplicate using an Applied Biosystems CFX96 Real-Time PCR Detection system (Chengdu BIO-BRI Science & Technology Co., Ltd). The expression of *PKCα*, *PKCβ*, *fga*, *fgb*, *fgg* and *vWF* were analyzed following the manufacturer’s protocol. The β-actin transcript was served as an internal control. The primer sequences (TsingKe) were provided in Additional file 1: Table S1. The experimental groups were normalized relative to the control group (100%) and the percentage values were then compared.

### Statistical analysis

All data were expressed as the mean  ±  SEM error. Statistical comparisons among groups were performed by one-way ANOVA followed by the least significant difference post hoc test (two-tailed). Statistical calculations were performed using IBM SPSS Statistics 21 software. For each test, at least three independent parallel experiments were performed.

## Results

### Analysis of DS–SQ extract by HPLC–ESI–MS/MS

DS–SQ (1:1) extract was analyzed by LC–MS. The results showed that the phenolic acids exhibited [M−H]^−^, [M+HCOOH]^−^ and [2 M–H]^−^ in the negative mode or [M+H]^+^ and [M+Na]^+^ in the positive mode; the diterpenoid quinones mainly showed [M+H]^+^and [M+Na]^+^ in the positive mode; the saponins exhibited [M–H]^−^ and [M+HCOOH]^−^ in the negative mode or [M+H]^+^ and [M+Na]^+^ in the positive mode. The phenolic acids and saponins were sensitive in both the positive and negative mode, while diterpenoid quinones were more sensitive in the positive mode. The total ion chromatography (TIC) of DS–SQ in the positive mode and negative mode were shown in Additional file 1: Fig. S1. By attentive study of the mass spectra of those components and comparison with standards and reference data [28–32], a total of thirty-three chemical components, including twelve phenolic acids, seven diterpenoid quinones and fourteen saponins, were tentatively identified (Additional file 1: Table S2).

### The anti-thrombotic activity of DS–SQ extract on PHZ-induced zebrafish thrombosis model

**Fig. 1:**
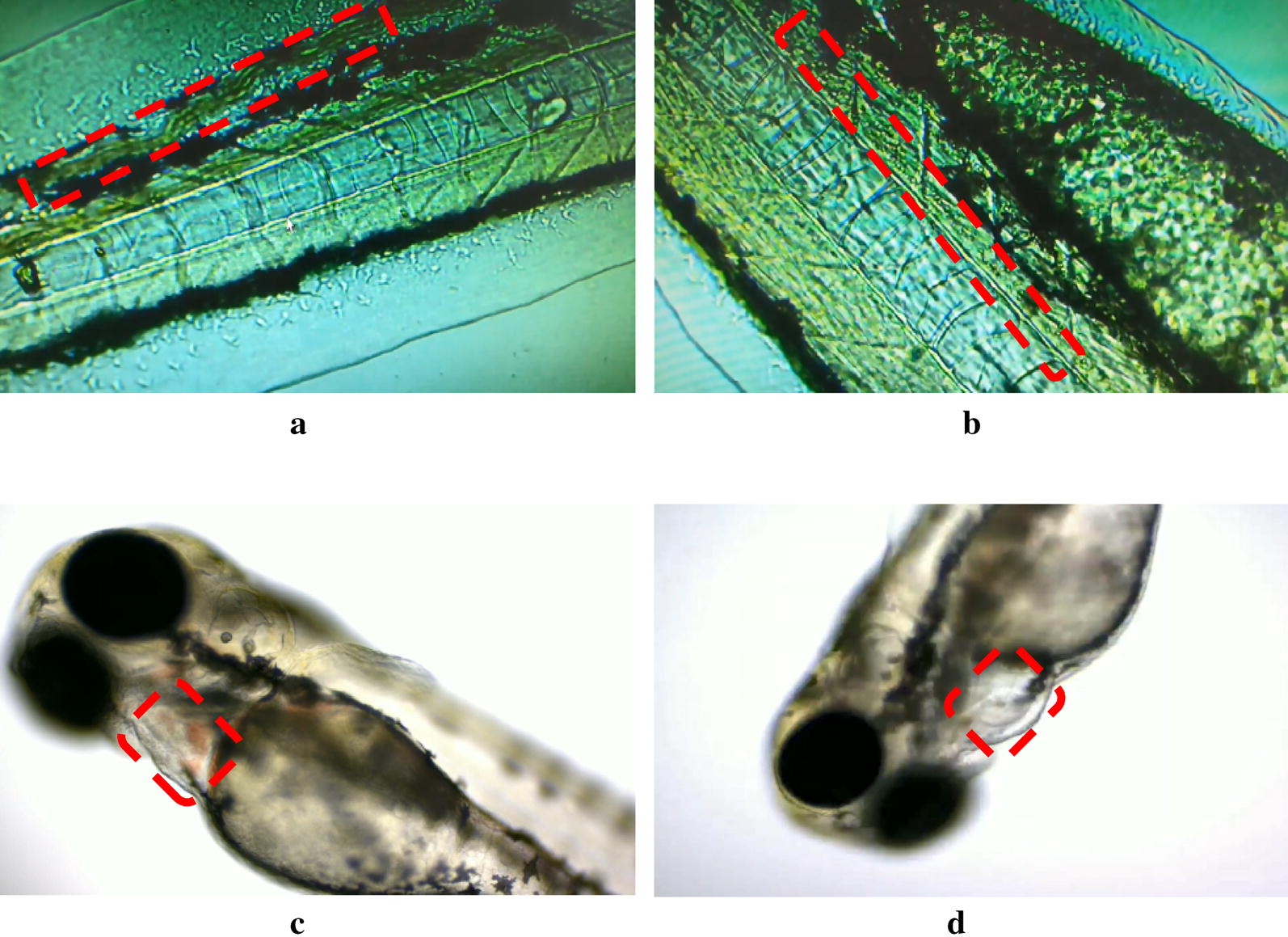
The blood velocity of control group (**a**) and model group (**b**) in caudal vein; and the blood flow of blank group (**c**) and model group (**d**) in heart (no dyeing)

**Fig. 2:**
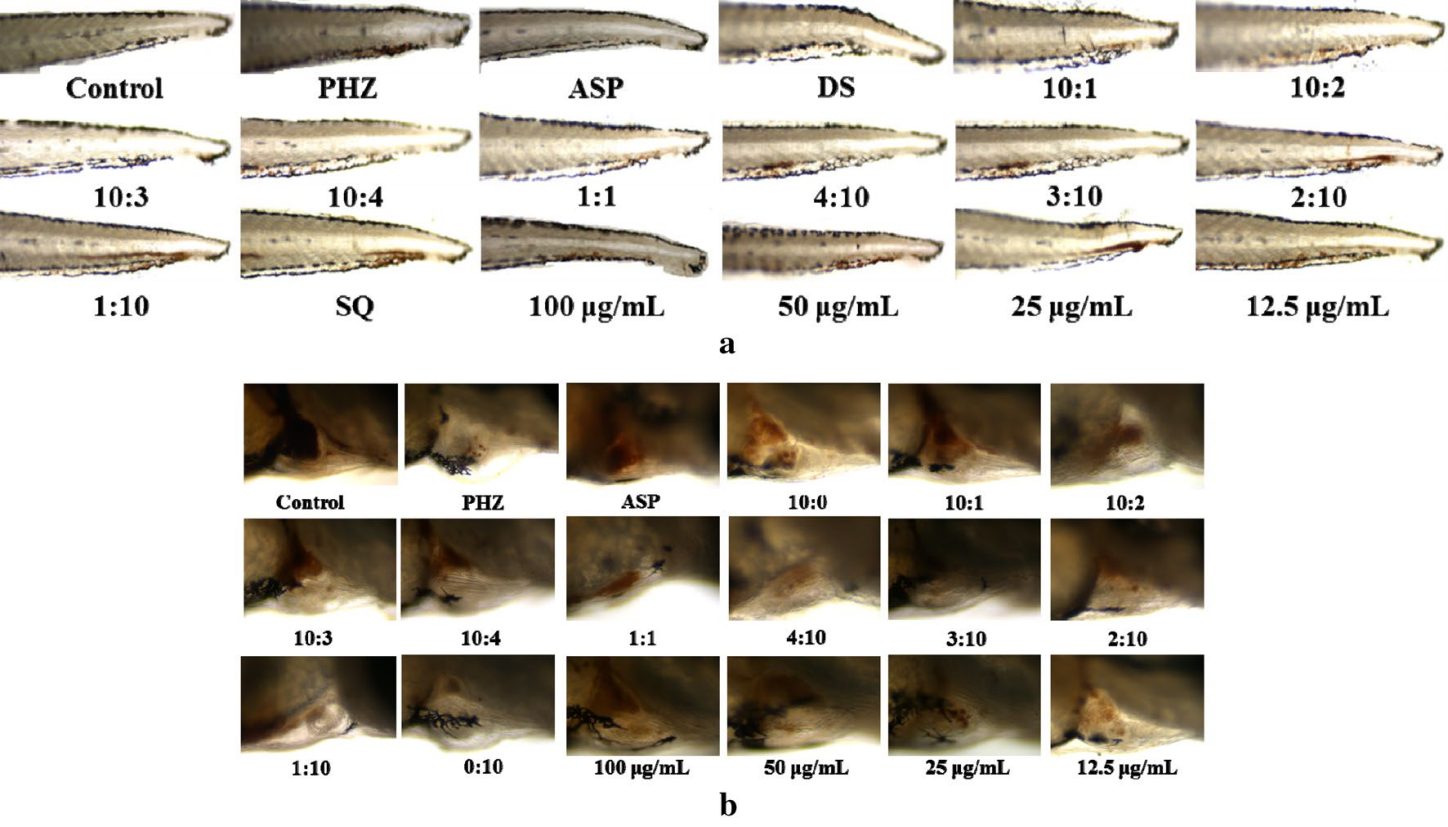
Erythrocytes aggregation in the caudal vein (**a**) and the thrombus staining area in the heart (**b**) of zebrafish larvae of control group, PHZ (1.5 μM), G1 (ASP, 25 μg/mL), G2–G12 (100 μg/mL of DS–SQ with different ratios) and G13–G16 groups (10:1 of DS–SQ with different concentrations)

Compared with the control group, the erythrocyte aggregation could be obviously observed in the caudal vein of the model (PHZ) group (Fig. 2a). However, the staining area of cardiac erythrocytes was significantly decreased (*P *< 0.001) (Fig. 2b). The blood flow velocity in the caudal vein of PHZ-induced model group was discontinuity and slow observed under the microscope (Fig. 1a, b), the blood in the heart decreased significantly (Fig. 1c, d). The above results indicated that the PHZ-induced zebrafish thrombosis model was successful developed.

**Fig. 3:**
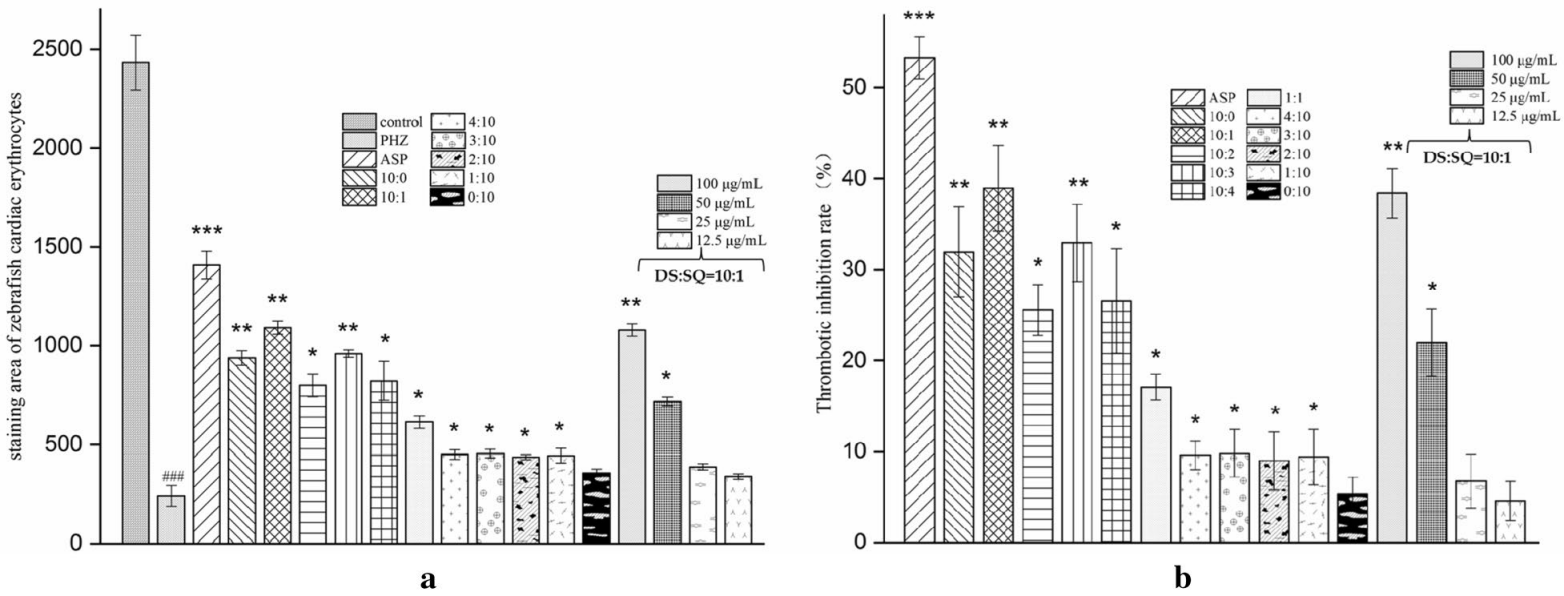
The staining area of cardiac erythrocytes (**a**) and the thrombotic inhibition percentage (**b**) of control group, PHZ (1.5 μM), G1 (ASP, 25 μg/mL), G2–G12 (100 μg/mL of DS–SQ with different ratios) and G13-G16 groups (10:1 of DS–SQ with different concentrations). Data are expressed as mean ± SD (n = 3), ^###^*P *< 0.001 versus the control group, *P < 0.05, **P < 0.01 and ***P < 0.001 versus the PHZ-induced model group

Compared with the model group, the staining area of cardiac erythrocytes increased (Fig. 2b) and the aggregation of erythrocyte in the caudal vein decreased (Fig. 2a) to different degrees in groups G1–G16. The thrombotic inhibition percentages (Fig. 3a, b) were 53.6% (*P *< 0.001) and 42.3% (*P *< 0.01) of group G1 and group G3, respectively, indicating that DS–SQ showed therapeutic effect on PHZ-induced zebrafish thrombus. When treated with 100 μg/mL of DS–SQ with the combination ratio of 10:1, it showed the best anti-thrombotic activity. From the thrombotic inhibition percentage of group G13–G16 (38.4%, 22.0%, 6.7%, 4.6%), it presented an obvious dose-dependent relationship.

**Fig. 4:**
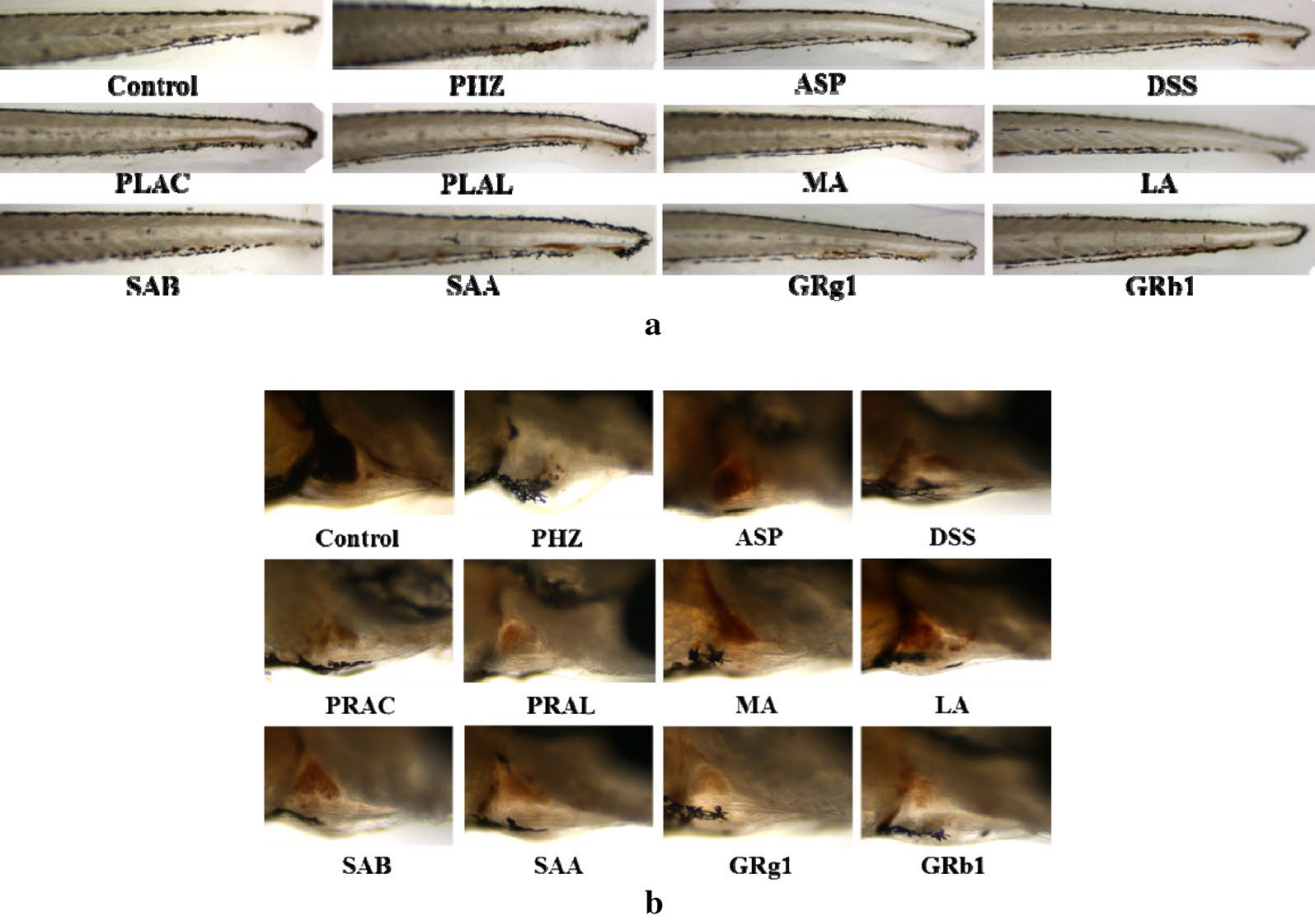
Erythrocytes aggregation in the caudal vein (**a**) and the thrombus staining area in the heart (**b**) of zebrafish larvae of control group, PHZ (1.5 μM), G1 (ASP, 25 μg/mL) and G17–G25 groups (25 μg/mL of DSS, PRAC, PRAL, MA, LA, SAB, SAA, GRg1 and GRb1, respectively)

**Fig. 5:**
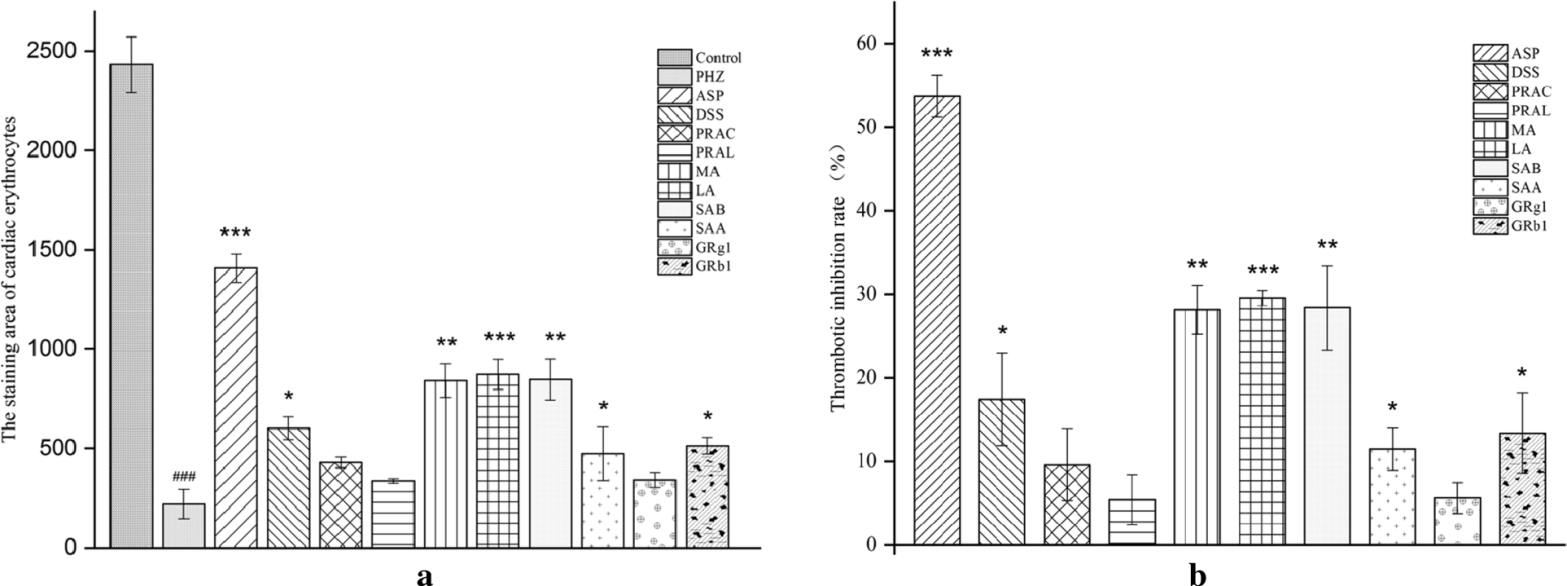
The staining area of cardiac erythrocytes (**a**) and the thrombotic inhibition percentage (**b**) of control group, PHZ (1.5 μM), G1 (ASP, 25 μg/mL), G17–G25 (25 μg/mL of DSS, PRAC, PRAL, MA, LA, SAB, SAA, GRg1 and GRb1, respectively). Data are expressed as mean ± SD (n = 3), ^###^*P *< 0.001 versus the control group, *P < 0.05, **P < 0.01 and ***P < 0.001 versus the PHZ-induced model group

In order to further explore the anti-thrombotic activity of components in DS–SQ herbal pair, DSS, PRAC, PRAL, MA, LA, SAB, SAA of DS and GRg1, GRb1 of SQ were selected, and PHZ-induced zebrafish thrombosis model was used to evaluated their anti-thrombotic activity. The results were shown in Figs. 4 and 5. MA, LA and SAB had the most significant effect (*P *< 0.01 and *P *< 0.001, respectively), the thrombosis inhibition percentage were 28.1%, 29.5% and 28.4%, respectively.

### DS–SQ decrease the PHZ-induced upregulation of *PKCα*, *PKCβ*, *fga*, *fgb*, *fgg* and *vWF* gene expression in zebrafish

**Fig. 6:**
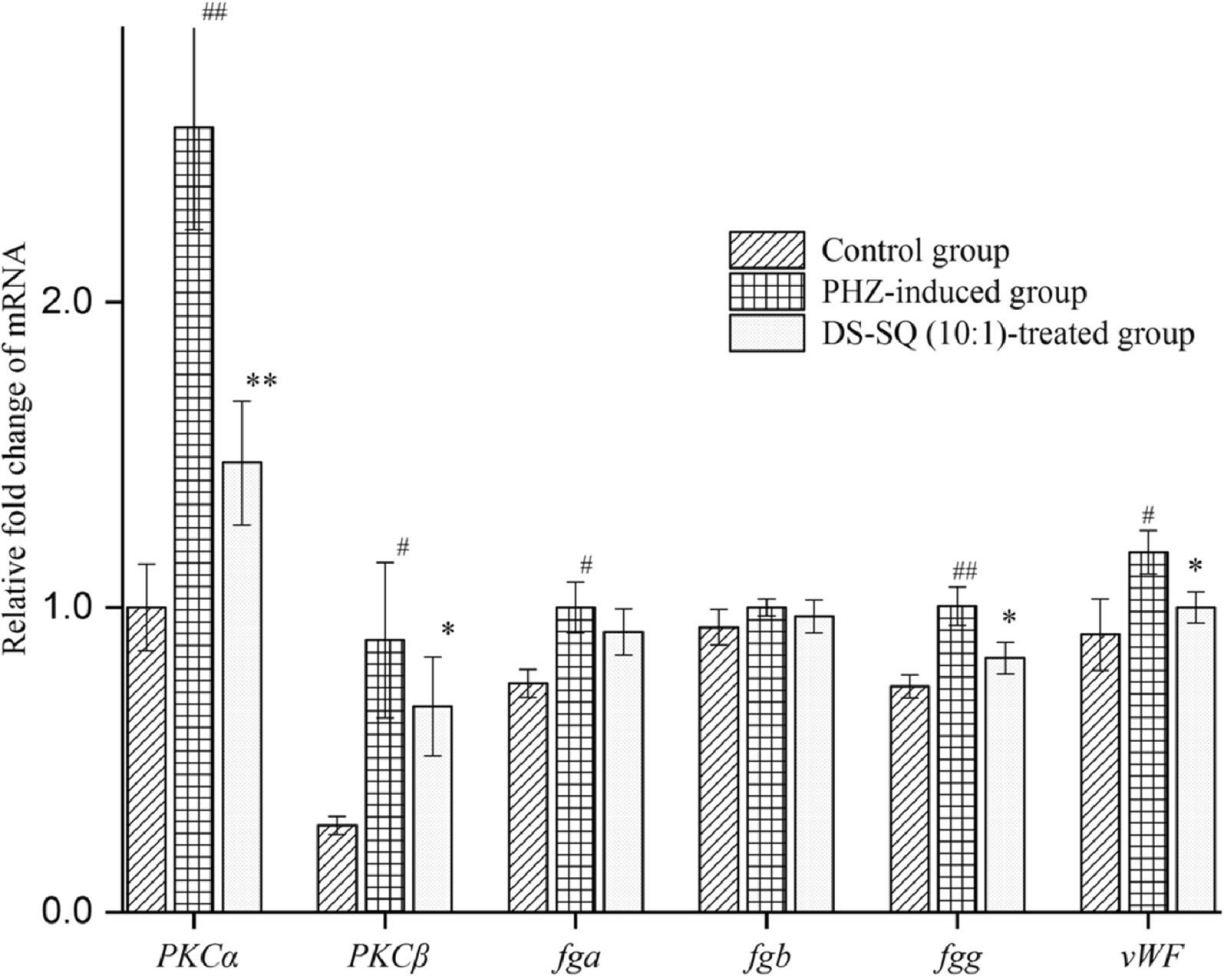
Gene expressions of control group, PHZ-induced model group (1.5 μM) and DS–SQ-treated group (100 μg/mL of DS–SQ with the ratio of 10:1). Data are expressed as mean ± SD (n = 3), ^#^*P *< 0.05 and ^##^*P *< 0.01 versus the control group, *P < 0.05 and **P < 0.01 versus the PHZ-induced group

In order to explore the possible anti-thrombotic mechanism of DS–SQ, quantitative analysis of thrombotic relating mRNA was carried out. After extracting the RNA of zebrafish, the purity and integrity of the RNA were assessed, the result of the agarose gel electrophoresis (Additional file 1: Fig. S3A) showed a clear band of light and shade without trailing and the absorbance ratios (Additional file 1: Fig. S3B) were stable at around 1.90, which indicated that the extracted RNA had high purity and integrity. As shown in Fig. 6, the mRNA expression of *PKCα*, *PKCβ*, *fga*, *fgb*, *fgg* and *vWF* were upregulated at different levels after incubating with PHZ, and the expression of genes involved in thrombosis were restored after treating with DS–SQ at the combination ratio of 10:1. These results indicated that the mechanism of anti-thrombotic effect of DS–SQ may be associated with the downregulation of the expression of *PKCα*, *PKCβ*, *fga*, *fgb*, *fgg* and *vWF*.

## Discussion

Cardiovascular endothelial injury and the changes of hemodynamics and blood coagulation function are the most important causes of thrombosis [33]. DS–SQ is known as a commonly used TCM for the treatment of cardiovascular diseases such as thrombosis for a long time. Pharmacological research revealed that DS–SQ has the effect of promoting blood circulation and antagonizing myocardial ischemia and reperfusion injury, can be used for the treatment of coronary heart disease and angina pectoris [34, 35]. Herein, the optimum compatibility proportion and the underlying action mechanism of DS–SQ on anti-thrombotic effect were further investigated in this study.

There were extensive studies on phytochemical of DS and SQ [29]. DS contains two types of major bioactive components, including water-soluble phenolic acids and fat-soluble diterpenoid quinones. As the major bioactive components, protopanaxadiol and protopanaxatriol saponins are abundant in SQ [28]. Many analytical methods have been developed for the component identification of the crude drug of DS and SQ. For example, Hong et al. developed an analytical method for the identification and quantification of six phenolic acids in *S. miltiorrhiza* [36]. Yuan et al. identified new trace triterpenoid saponins from the roots of *P. notogiseng* by LC–ESI–MS/MS [31]. In the present study, thirty-three chemical compounds, including twelve phenolic acids, seven diterpenoid quinones and fourteen saponins were identified by LC–ESI–MS/MS from the extract of DS–SQ. It was reported that protocatechuic aldehyde and salvianolic acid A, as the water-soluble phenolic acids of DS, and 15, 16-dihydrotanshinone I, cryptotanshinone and tanshinone IIA, as the lipophilic diterpenoid quinones of DS, showed the direct thrombin inhibition activities [37–39]. While, GRg1, as a high content saponin in SQ, could inhibit platelet activation via the inhibition of PKC and ERK pathway and attenuate arterial thrombus formation in vivo [40].

PHZ can promote vascular endothelial injury and platelets aggregation, which eventually result in the formation of thrombosis [41]. In the present study, a PHZ-induced thrombosis model in zebrafish was established. On the other hand, ASP can inhibit arachidonic acid release in platelets and reduce the production of thromboxane A2 by inhibiting the effects of epoxidase, peroxidase and thromboxane synthase, thereby inhibiting the adhesion and aggregation of platelets and preventing blood coagulation and thrombosis [42]. Therefore, ASP was selected as a positive control drug in the present study. Results indicated that the thrombus could be significantly decreased after the treatment of ASP. Furthermore, DS–SQ at the ratios of 10:1 decreased the PHZ-induced thrombosis in a dose-dependent manner, which indicated that there is a significant synergistic effect in anti-thrombotic activity of DS–SQ herbal pair. However, these two herbs are mixed together before decoction in practical application, which is different from the present study. Therefore, further study can be focused on the impact of preparation methods on the chemical components in DS–SQ and its anti-thrombotic activity. Meanwhile, MA, LA and SAB from DS also showed an anti-thrombotic effect. It is speculated that DS may play a major role in inhibiting thrombosis [43]. However, SQ showed a dual efficacy in hemostasis [44] and anti-thrombosis [45].

Subsequently, the possible anti-thrombotic molecular mechanism of DS–SQ was further studied. The protein kinase C family of serine/threonine kinases plays a well-established and critical role in platelet function and thrombosis [46]. Both of *PKCα* and *PKCβ* are two conventional PKC isoforms expressed in platelets. It was reported that *PKCα* is an important positive regulator of platelet function and a crucial regulator of thrombosis in vitro and in vivo. Likewise, *PKCβ* showed the functions of regulation the outside-in signaling through the α_IIb_β_3_ integrin and positive regulation of thrombosis in vivo [47, 48]. The coagulation cascade is a series of sequential reactions of limited proteolysis of protein factors resulting in generation of thrombin, while fibrinogen, which is encoded by *fga*, *fgb* and *fgg* genes, is clearly one of the key proteins in coagulation cascade [49]. On the one hand, fibrinogen acts as the precursor of the fibrin net that gives structure to blood clots, on the other hand, it acts as the promoter of platelet aggregation and fibrinolysis [50]. The *vWF*, which is an adhesive multimeric glycoprotein that is crucial for the hemostasis, is synthesized in both endothelial cells and megakaryocytes. Previous report showed that *vWF* aggravates thrombotic disease such as stroke via a GPIb-dependent mechanism [51]. The result of RT-qPCR indicted that PHZ could upregulate the mRNA expression of *PKCα*, *PKCβ*, *fga*, *fgb*, *fgg* and *vWF*, while the expression quantity were significantly restored after the treatment of DS–SQ extract. These results indicated that the underlying anti-thrombotic mechanism of DS–SQ is related to the regulation of mRNA expression of these genes.

## Conclusion

In summary, thirty-three components were identified in DS–SQ by HPLC–ESI–MS/MS analysis. On the PHZ-induced zebrafish thrombosis model, DS–SQ at the combination ratio of 10:1 showed a significant synergistic effect in anti-thrombotic activity with a dose-dependent manner. Furthermore, MA, LA and SAB showed good thrombosis inhibitory activity, indicating that they may be the primary anti-thrombotic active compounds in DS, which further confirmed that DS played a major anti-thrombotic role in the DS–SQ herbal pair. In addition, the anti-thrombotic effect of DS–SQ may through the downregulation of *PKCα*, *PKCβ*, *fga*, *fgb*, *fgg* and *vWF* expressions.

## Supplementary information

**Additional file 1: Table S1.** Zebrafish primers used for RT-qPCR analysis. **Table S2.** Identification of the components of DS–SQ by HPLC–MS/MS. **Fig. S1.** Total ion chromatograms (TICs) of DS–SQ extract in both positive ion mode (A) and in negative ion mode (B). **Fig. S2.** The chemical structures of nine compounds from DS–SQ. **Fig. S3.** The results of agarose gel electrophoresis (A) and absorbance ratios of groups G28-G30 (B).

## Data Availability

The research data generated from this study is included within the article.

## Abbreviations

ACN: Acetonitrile
ASP: Aspirin
CDL: Curved desolvation line
CID: Collision induced dissociation
DMSO: Dimethylsulfoxide
DS: *Salvia miltiorrhiza*
DSS: Danshensu
DS–SQ: Danshen–Sanqi herbal pair
ESI–MS: Electrospray ionization-mass spectrometry
FA: Formic acid
GRb1: Ginsenoside Rb1
GRg1: Ginsenoside Rg1
LA: Lithospermic acid
LC–MS/MS: Liquid chromatography/tandem mass spectrometry
MA: Rosmarinic acid
NCDs: Noncommunicable diseases
PHZ: Phenylhydrazine
PRAC: Protocatechuic acid
PRAL: Protocatechualdehyde
RT-qPCR: Real-time quantitative PCR
TCMs: Traditional Chinese medicines
TIC: Total ion chromatography
SAA: Salvianolic acid A
SAB: Salvianolic acid B
SQ: *Panax notoginseng*
