# Supplementary material for: Antithrombotic effect and action mechanism of Salvia miltiorrhiza and Panax notoginseng herbal pair on the zebrafish
Source: Chin Med. 2020 Apr 16;15:35. doi: 10.1186/s13020-020-00316-y (PMC7164150; doi:10.1186/s13020-020-00316-y)
Supplement: Supplementary file 1 — Additional file 1: Table S1. Zebrafish primers used for RT-qPCR analysis. Table S2. Identification of the components of DS–SQ by HPLC–MS/MS. Fig. S1. Total ion chromatograms (TICs) of DS–SQ extract in both positive ion mode (A) and in negative ion mode (B). Fig. S2. The chemical structures of nine compounds from DS–SQ. Fig. S3. The results of agarose gel electrophoresis (A) and absorbance ratios of groups G28-G30 (B). [file 13020_2020_316_MOESM1_ESM.docx]

**Additional file**

**Antithrombotic effect and the action mechanism of** ***Salvia miltiorrhiza* and *Panax notoginseng* herbal pair on zebrafish**

Shi-Jun Yin ^a^, Ying-Qing Luo ^b^, Cong-Peng Zhao ^a^, Hua Chen ^a^, Zhang-Feng Zhong ^c^, Sheng-Peng Wang ^c^, Yi-Tao Wang ^c**^, Feng-Qing Yang ^a*^

^a^ School of Chemistry and Chemical Engineering, Chongqing University, Chongqing 401331, China

^b^ School of Life Sciences, Chongqing University, Chongqing 401331, China

^c^ State Key Laboratory of Quality Research in Chinese Medicine, Institute of Chinese Medical Sciences, University of Macau, Macao, China

*****Corresponding author:

Prof. Dr. Feng-Qing Yang, School of Chemistry and Chemical Engineering, Chongqing University, Chongqing 401331, China. E-mail: fengqingyang@cqu.edu.cn. Phone number: +8613617650637.

Prof. Yi-Tao Wang, State Key Laboratory of Quality Research in Chinese Medicine, Institute of Chinese Medical Sciences, University of Macau, Macao, China. E-mail: ytwang@um.edu.mo.

**Table of contents**

**Supplementary Tables**

**Supplementary Figures**

**Table S1.** Zebrafish primers used for RT-qPCR analysis

**Table S2.** Identification of the components of DS-SQ by HPLC-MS/MS

**Fig. S1.** Total ion chromatograms (TICs) of DS-SQ extract in both positive ion mode (A) and in negative ion mode (B)

**Fig. S2.** The chemical structures of nine compounds from DS-SQ

**Fig. S3.** The results of agarose gel electrophoresis (A) and absorbance ratios of groups G28-G30 (B)

**Table S1.** Zebrafish primers used for RT-qPCR analysis

| Gene | Forward primer sequence (5′ → 3′) | Reverse primer sequence (5′ → 3′) |
| --- | --- | --- |
| *PKCα* | 5′ TCGTTGCTTTGTGTATCAGCCATTG 3′ | 5′ ACCCCCTGATGAAGAGAAGAGAGAA 3′ |
| *PKCβ* | 5′ CCGGCTCTGCCATTCTAAAAGCGGG 3′ | 5′ ACAAAGCAGCAGACTGAAAGAAGGA 3′ |
| *fga* | 5′ GCAAGTTTCCCACATCAGGT 3′ | 5′ GTCGGGCATATCTTCTTCCA 3′ |
| *fgb* | 5′ CAAGGAGTGCGAAGACATCA 3′ | 5′ GTATTTCCAGCCGTTCCTGA 3′ |
| *fgg* | 5′ TTGGACGTGGATGGACTGTA 3′ | 5′ GTGTCCCTGGAACTTGTCGT 3′ |
| *vWF* | 5′ CTCCGTTTGACCGCAAAA 3′ | 5′ ACAGCAGGTGTCTCCGATCT 3′ |
| β-action | 5′ ATGCCCCTCGTGCTGTTTT 3′ | 5′ TCTGTCCCATGCCAACCAT 3′ |

**Table S2.** Identification of the components of DS-SQ by HPLC-MS/MS

| **Peak**  **No.** | **Retention time**  **t_R_ (min)** | **Molecular weight**  **(MW)** | **MS (*m/z*)** | **Identification** | **Source** |
| --- | --- | --- | --- | --- | --- |
| 1 | 13.347 | 198 | 221 ([M+Na]^+^); 395 ([2M-H]^-^) | Danshensu | DS |
| 2 | 22.587 | 194 | 193 ([M-H]^-^) | Caffeic acid methyl ester | DS |
| 3 | 24.140 | 180 | 359 ([2M-H]^-^) | Caffeic acid | DS |
| 4 | 25.051 | 418 | 441 ([M+Na]^+^) | Salvianolic acid D | DS |
| 5 | 36.563 | 963 | 1008 ([M+HCOOH]^-^) | Notoginsenoside R3/R6 | SQ |
| 6 | 38.103 | 963 | 1008 ([M+HCOOH]^-^) | Notoginsenoside R3/R6 | SQ |
| 7 | 39.540 | 932 | 955 ([M+Na]^+^), 775 ([M+Na-glc-H_2_O]^+^); 977 ([M+HCOOH]^-^) | Notoginsenoside R1 | SQ |
| 8 | 41.080 | 538 | 561 ([M+Na]^+^); 537 ([M-H]^-^) | Lithospermic acid | DS |
| 9 | 41.593 | 800 | 823 ([M+Na]^+^); 845 ([M+HCOOH]^-^), 799 ([M-H]^-^),  637 ([M-H-glc]^-^), 475 ([M-H-2glc]^-^) | Gensenoside Rg1 | SQ |
| 10 | 43.441 | 360 | 359 ([M-H]^-^) | Rosmarinic acid | DS |
| 11 | 44.160 | 947 | 992 ([M+HCOOH]^-^), 946 ([M-H]^-^) | Gensenoside Re | SQ |
| 12 | 46.624 | 718 | 741 ([M+Na]^+^), 740 ([M+Na-H]^+^), 561 ([M+Na-CA]^+^), 517 ([M+Na-CA-CO_2_]^+^),  362 ([M+Na-CA-DSS-H]^+^), 319 ([M+Na-CA-DSS-CO_2_]^+^); 717 ([M-H]^-^) | Salvianolic acid B | DS |
| 13 | 48.575 | 718 | 741 ([M+Na]^+^); 717 ([M-H]^-^) | Salvianolic acid E | DS |
| 14 | 51.449 | 732 | 731 ([M-H]^-^) | 9’-methyl lithospermate B | DS |
| 15 | 51.552 | 494 | 493 ([M-H]^-^) | Salvianolic acid A | DS |
| 16 | 53.569 | 492 | 493 ([M+H]^+^), 277 ([M+H-DSS-H_2_O]^+^); 491 ([M-H]^-^), 229 ([M-H-DSS-CO-2H_2_O]^-^) | Salvianolic acid C | DS |
| 17 | 54.208 | 566 | 589 ([M+Na]^+^) | Ethyllithospermate | DS |
| 18 | 56.069 | 800 | 845 ([M+HCOOH]^-^), 799 ([M-H]^-^) | Gensenoside Rf | SQ |
| 19 | 56.377 | 770 | 815 ([M+HCOOH]^-^) | Notoginsenoside R2 | SQ |
| 20 | 58.533 | 1109 | 1131 ([M+Na]^+^), 787; 1154 ([M+HCOO]^-^) | Gensenoside Rb1 | SQ |
| 21 | 59.868 | 638 | 683 ([M+HCOO]^-^) | Gensenoside F1/Rh1 | SQ |
| 22 | 60.792 | 784 | 785 ([M+H]^+^); 783 ([M-H]^-^) | Gensenoside Rg2 | SQ |
| 23 | 61.613 | 638 | 683 ([M+HCOO]^-^) | Gensenoside F1/Rh1 | SQ |
| 24 | 65.515 | 946 | 969 ([M+Na]^+^), 789 ([M+Na-glc-H_2_O]^+^); 991 ([M+HCOOH]^-^) | Gensenoside Rd | SQ |
| 25 | 70.340 | 946 | 969 ([M+Na]^+^); 991 ([M+HCOOH]^-^) | Notoginsenoside K | SQ |
| 26 | 75.152 | 784 | 785 ([M+H]^+^); 829 ([M+HCOOH]^-^) | Gensenoside F2 | SQ |
| 27 | 89.115 | 312 | 313 ([M+H]^+^) | Tanshindiol A | DS |
| 28 | 94.656 | 296 | 319 ([M+Na]^+^), 263 ([M+Na-2CO]^+^), 123 | Cryptotanshinone | DS |
| 29 | 98.252 | 276 | 277 ([M+H]^+^), 201 ([M+H-H_2_O-CO-2CH_3_]^+^), 179, 157 ([M+H-H_2_O-CO-2CH_3_-CO_2_]^+^) | Tanshinone I | DS |
| 30 | 99.587 | 278 | 279 ([M+H]^+^), 201 ([M+H-H_2_O-CO-OH-CH_3_]^+^), 145,  121 ([M+H-3H_2_O-CO-OH-CH_3_-CO_2_]^+^) | Methylene tanshiqunone | DS |
| 31 | 105.233 | 294 | 295 ([M+H]^+^), 193 ([M+H-CO_2_-CO-2CH_3_]^+^) | Tanshinone IIA | DS |
| 32 | 107.282 | 282 | 305 ([M+Na]^+^), 118 | Miltirone | DS |
| 33 | 108.327 | 280 | 325 ([M+HCOOH]^-^) | Danshenxinkun B | DS |

DS, danshen; SQ, sanqi; glc, β-D-glucopyranosyl; CA, caffeic acid; DSS, Danshensu


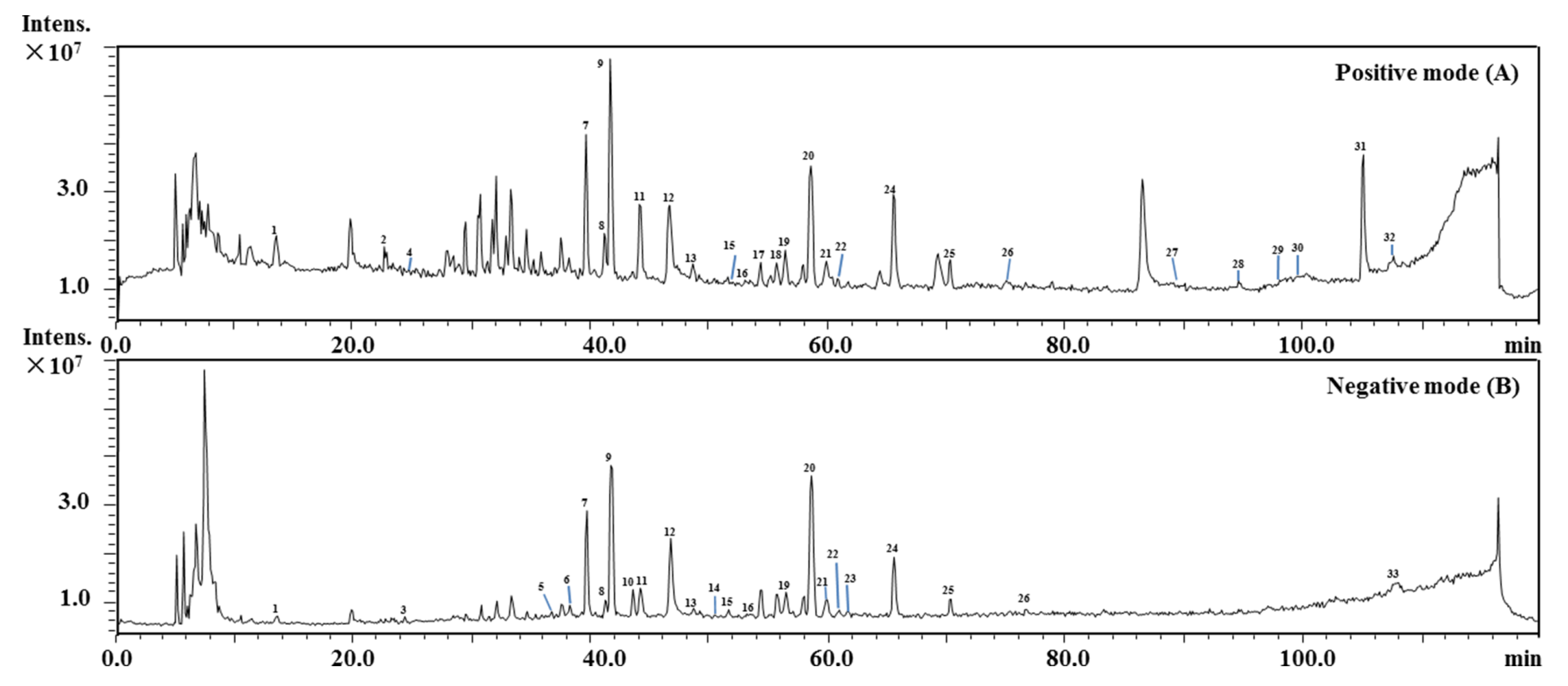


**Fig. S1.** Total ion chromatograms (TICs) of DS-SQ extract in both positive ion mode (A) and in negative ion mode (B)

**
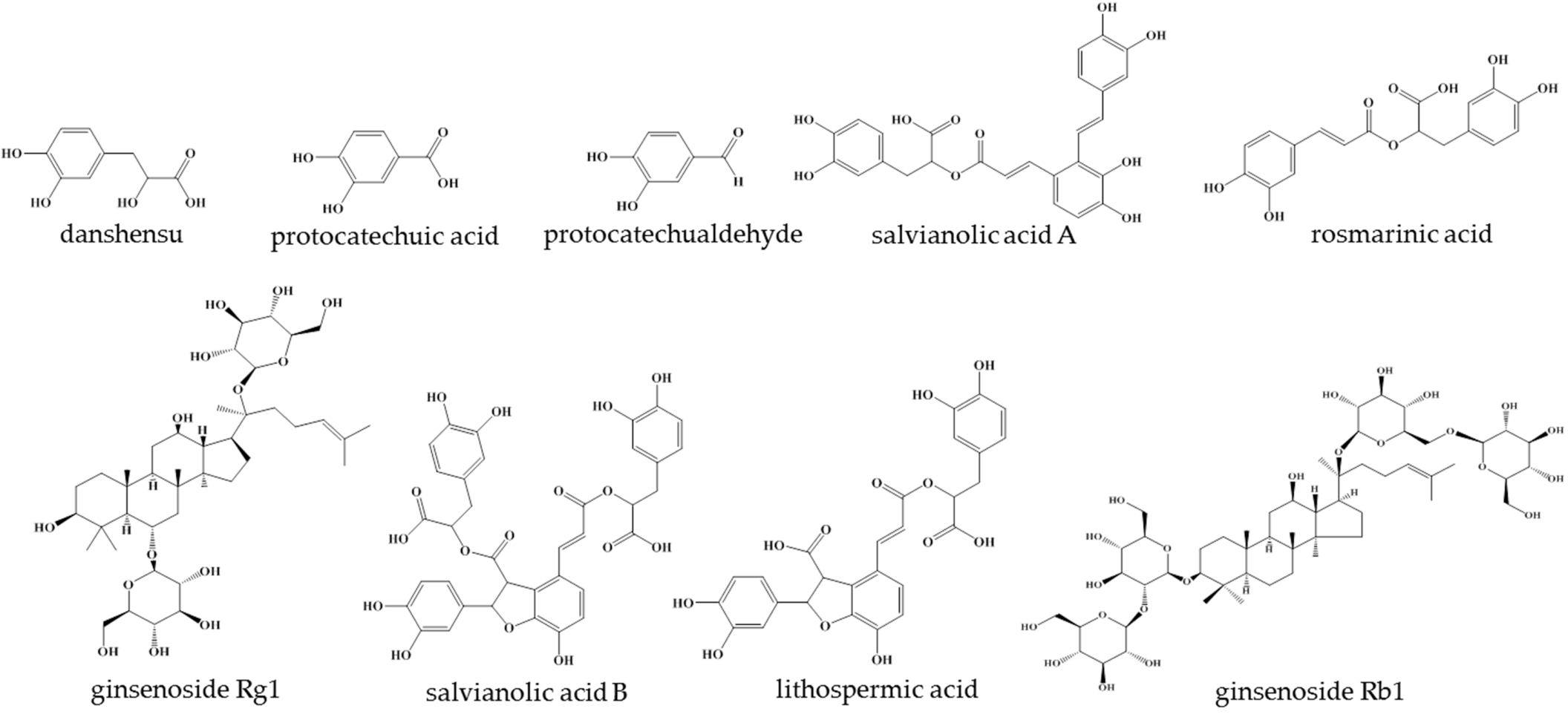
**

**Fig. S2.** The chemical structures of nine compounds from DS-SQ


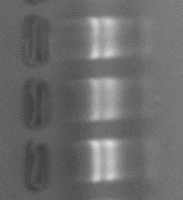


Control group →

PHZ-induced group →

DS-SQ (10:1)-treated group →

(A)

|  | Control group | PHZ group | DS-SQ (10:1) group |
| --- | --- | --- | --- |
| OD260/OD280 | 1.91 | 1.93 | 1.90 |

**Note:** OD_260_/OD_280_ 1.8-2.2 showed high purity of RNA, less than 1.8 showed DNA and protein residue, less than 1.6 showed excessive drying of RNA

(B)

**Fig. S3.** The results of agarose gel electrophoresis (A) and absorbance ratios of group G28-G30 (B)
